# Supplementary material for: The Relationship between Mastitis and Antimicrobial Peptide S100A7 Expression in Dairy Goats
Source: Vet Sci. 2023 Nov 14;10(11):653. doi: 10.3390/vetsci10110653 (PMC10674537; doi:10.3390/vetsci10110653)
Supplement: Supplementary file 1 [file vetsci-10-00653-s001.zip › vetsci-2653959-supplementary.pdf]

Supplemental Table 1. Ingredients and chemical composition of the postpartum diets on the dry matter (DM) basis

Ingredient (% of DM)	Postpartum	Nutrient composition	Postpartum
Alfalfa hay	18.42	DM (% of fresh)	48.20
Corn	23.16	Neutral detergent fiber (NDF, %)	37.70
Wheat bran	8.37	Acid detergent fiber (ADF, %)	17.40
Soybean meal	9.02	Crude protein (CP, %)	16.40
Wheat straw	0.00	Starch (%)	25.40
Corn Silage	30.66	Ether extract (%)	3.20
Corn germ meal	3.26	Calcium (Ca, %)	0.66
Cottonseed meal	5.12	Phosphorus (P, %)	0.37
Calcium hydrophosphate	0.51	Magnesium (Mg, %)	0.19
Limestone	0.46	Sulfur (S, %)	0.20
Sodium carbonate	0.38	Chloride (Cl, %)	0.25
Sodium chloride	0.45	DCAD (mEq/kg of DM)[2]	+733
Mineral and vitamin premix [1]	0.19	NE_L (Mcal/kg)[3]	1.62

[1] The mineral-vitamin premix provided the following per kg of diets: vitamin A 250,000 IU, vitamin D 23,250 IU, vitamin E 1500 IU, manganese 800 mg, zinc 1800 mg, copper 370 mg, iron 2200 mg, cobalt 50 mg, iodine 30 mg, selenium 30 mg.

[2] The dietary cation-anion difference (DCAD) was calculated using the formula DCAD (mEq/kg of DM) = (Na + K) − (Cl^{++-} + S^{2-}).

[3] Net energy for lactation (NE_L) was estimated using CPM-Dairy software (version 3.0.8.1).

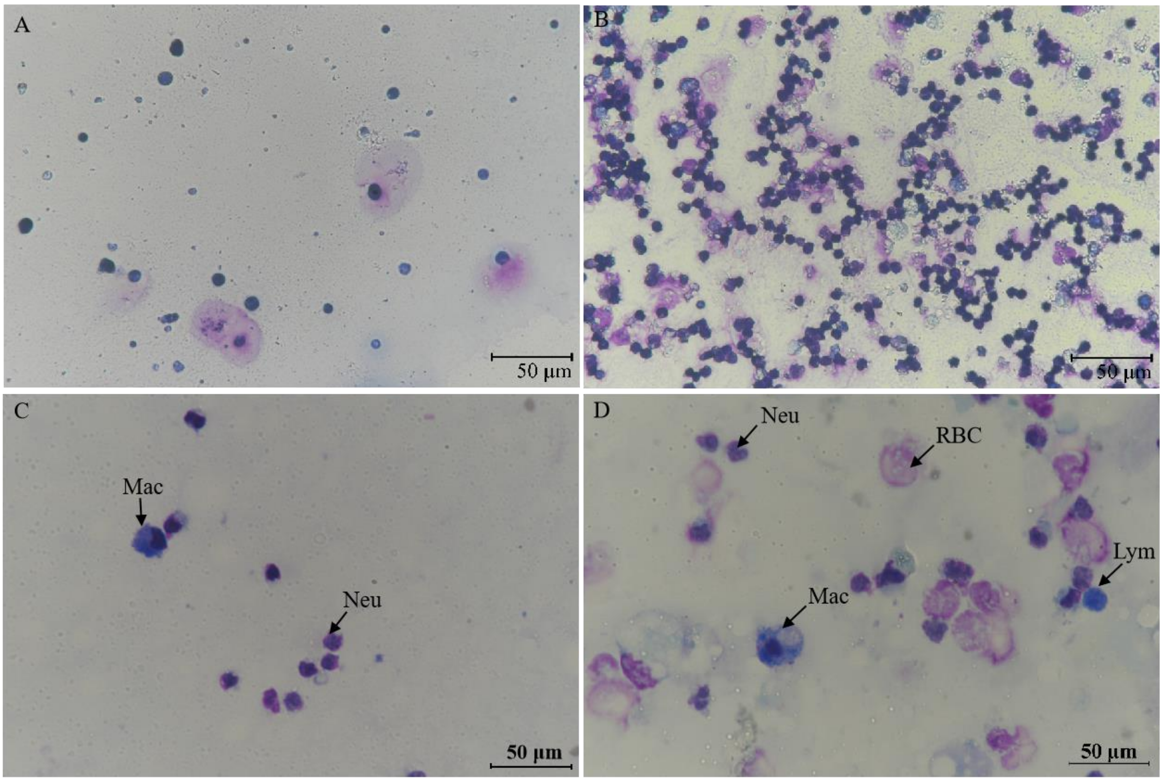

Figure S1. Stain of somatic cells in healthy dairy goat and mastitis dairy goat. (A,C) The represented images of healthy dairy goat milk somatic cells stain; (B,D) the represent images of mastitis dairy goat milk somatic cells stain; SCC in healthy and mastitis goat was 4.4 × 105 and 7.238 × 106 cells/mL, respectively. Mac = macrophages; Lym = lymphocytes; Neu = neutrophils; RBC = red blood cell. Scale bar = 50 μm.
